# 

**Published:** 1890-11-15

**Authors:** 


[Registered at the General Post Office as a Newspaper for transmission in the United Kingdom and Abroad.]

WITH EXTRA NURSING SUPPLEMENT

Per Annum, 6s. 6d.; post free.

Monthly Parts, 6d.; post free 8d.

Ditto per Annum, 8s.; post free.

### THE HOSPITAL

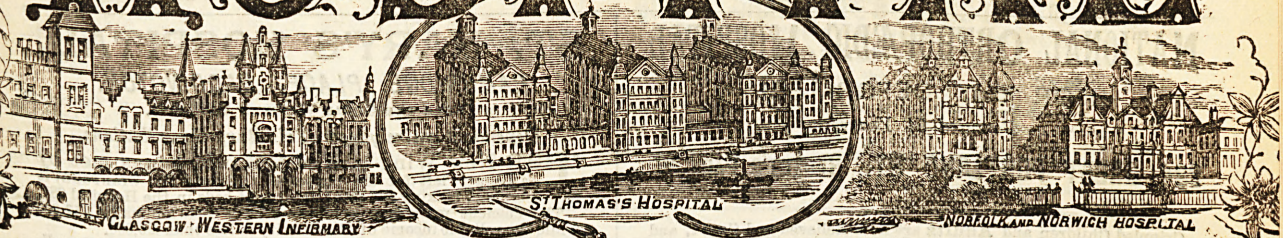

A WEEKLY JOURNAL OF

### Science, Medicine, Nursing and Philanthropy.

### SATURDAY, NOVEMBER 15th, 1890.

Diphtheria at Public Schools - 101
Passing Topics.—Tattlers - 102
Medical Schools in the United States - 103
The Relation of Modern Pathology and Pathological Anatomy to Practical Medicine - 104
The Doctor's Armchair.—Mrs. Fawcett and Socialism - 105
The Hertford British Hospital - 106
Everybody's Page - 107
Notes and News - 108
The Story of the Insane from Year to Year - 109
Annotations.—Koch's Cures and Other People's—Medical Racks and Thumbscrews—Religion Obligé! - 110
November - 114
The Practitioner's Mirror and Retrospect:—
Medicine—Iodides and Salicylates—Hemiatrophia Facialis —Weil's Disease—Menthol in Diphtheria - 111
Surgery—Flat-foot - 112
The London Post-Graduate Course at the Paddington Infirmary—Pathology - 112
New Drugs, Appliances, and Things Medical - 114
Scraps and Gleanings - 115
The Student of Medicine.—Women Students and Medical Examinations—Men of the Future (*with Portrait*)—Notes from the Schools—The Student's Bookshelf—Students' Medical Societies—Scholarships and Prizes—Pass Lists, &c. 116
General Charities.

### EXTRA SUPPLEMENT—THE NURSING MIRROR.

En Passant.—Bridport Nurse Society—Dundee Nurses—Belfast Nurses—Croydon Institute—Taunton Victoria Institute—Ladies on Committees—To Our Correspondents xxix
Lectures on Surgical Ward Work and Nursing. By Alex. Miles, M.B. (Edin.), C.M., F.R.C.S.E.—Lecture III.—Antiseptics in Common Use xxx
A District Nurse's Case Book xxxi
Words of Consolation.—Love xxxi
Nurses on Their Travels.—Johannesburg Again - xxxii
Everybody's Opinion.—Cottage Nurses—Male Nurses xxxii
Death in Our Ranks - xxxiii
Appointments—Examination Questions - xxxiii
Notes and Queries—Vacancies - xxxiii
Theatricals—Lectures - xxxiv
"A Voice from the Children" - xxxiv
Amusements and Relaxation - xxxiv

